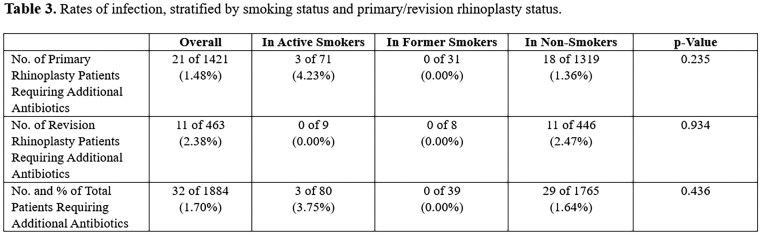# Evaluating the Safety of Rhinoplasty in Smokers

**DOI:** 10.1093/asjof/ojaf018.018

**Published:** 2025-05-13

**Authors:** Bugra Tugertimur, Shaishav Datta, Paige Goote, Matthew Morris, Alannah Phelan, Alexia Lucas, Jaime Bernstein, Steven Hanna, David Mattos, Richard Reish

**Affiliations:** Manhattan Eye, Ear and Throat Hospital, New York, NY; New York Plastic Surgical Group, New York, NY; University of Toronto, Toronto, ON, Canada; Manhattan Eye, Ear and Throat Hospital, New York, NY; New York Plastic Surgical Group, New York, NY; Manhattan Eye, Ear and Throat Hospital, New York, NY; New York Plastic Surgical Group, New York, NY; Manhattan Eye, Ear and Throat Hospital, New York, NY; Private Practice, Toronto, ON, Canada; New York Plastic Surgical Group, New York, NY; Manhattan Eye, Ear and Throat Hospital, New York, NY

## Abstract

**Goals/Purpose:**

Smoking negatively impacts tissue perfusion and wound healing, raising concerns about infection and delayed recovery in surgical patients. While smoking remains a strict contraindication in some procedures with extensive dissection, such as abdominoplasty and facelift surgery, the risks of smoking in rhinoplasty patients are not well known. Given the nasal region's robust vascular supply, the risk of smoking complications may be less in rhinoplasty. This study explores whether smoking should be considered a contraindication for rhinoplasty by comparing postoperative infection rates and the need for revision surgery between smokers and nonsmokers. Using a decade of patient data, we aim to assess whether there is an increased risk of infection or revision surgery in smokers. The findings will provide valuable insights to guide plastic surgeons in making informed decisions and ensuring safe, successful outcomes for both smokers and nonsmokers.

**Methods/Technique:**

A retrospective review was conducted on the senior author’s (R.G.R.) rhinoplasty cases from July 2014 to June 2022, including all patients treated in this period. The study was approved by the BRANY Institutional Review Board. All patients underwent open rhinoplasty under general anesthesia, prioritizing septal cartilage for reconstruction. If septal cartilage was inadequate, fresh frozen cartilage (FFCC) from MTF Biologics was used; no alloplastic materials were utilized.

Patients were categorized as active smokers, former smokers, and non-smokers. Active smokers used any inhaled tobacco products (e.g., cigarettes, cigars, vaping) within 4 weeks before and/or after surgery. Former smokers had quit over 4 weeks prior to surgery with no intent to resume, and non-smokers had no history of tobacco use. Patients with less than one-year follow-up were excluded. After reviewing 2003 cases of rhinoplasty, 1884 patients were found to match both the inclusion and exclusion criteria.

Patient demographics and surgical outcomes were collected through manual chart review. Primary outcomes included infection and revision rates, with infections identified by clinical signs requiring antibiotics or further intervention post-prophylactic antibiotics. Revision rhinoplasty was defined as any subsequent open procedure. Infection and revision rates were compared across active smokers, former smokers, and non-smokers, with subgroups for primary and revision rhinoplasty patients.

**Results/Complications:**

A total of 1884 patients consisting of 1673 (88.80%) females and 211 (11.20%) males met inclusion criteria with an average age of 30.7 years and BMI of 22.47 kg/m2. Among these patients, 1421 (75.42%) were primary rhinoplasty cases and 463 (41.5%) were revisions. The average length of follow-up was 23.8 months. This study’s rhinoplasty patient population consists of 81 (4.30%) active smokers, 38 (2.02%) former smokers, and 1765 (93.68%) non-smokers (Table 1).

In our patient population, we included patients who underwent both primary and revision rhinoplasty. In the overall population, there were 62 (3.29%) patients that underwent subsequent revision. 36 of 1421 (2.53%) of these patients belonged to the primary rhinoplasty group and 26 of 463 (5.62%) to the revision rhinoplasty population. In comparison, revisions were performed on 3 of 80 (3.75%) active smokers, 1 of 39 (2.56%) former smokers, and 58 of 1,765 (3.29%) non-smokers. Among these groups, all 3 of 71 (4.23%) revision patients were among primary rhinoplasty patients in the active smoker population, whereas in the non-smoker population, the distribution was 32 (2.42%) for primary and 26 (5.83%) for revision cases. No statistically significant difference was observed between groups (Table 2).

Overall, 32 of 1884 (1.70%) of patients required 5-7 days of additional postoperative antibiotics for cellulitis. This was in addition to the standard postoperative antibiotic prophylaxis. 21 of 1421 (1.48%) of these patients belonged to the primary rhinoplasty group and 11 of 463 (2.38%) to the revision rhinoplasty patient population. 3 of 80 (3.75%) of patients requiring additional postoperative antibiotics were active smokers and 29 of 1765 (1.64%) were non-smokers. There was no incidence of use of additional postoperative antibiotics in the former smoker rhinoplasty population. Again, no statistically significant differences were found among the groups (Table 3).

**Conclusion:**

The results of this study indicate that active smoking should not be considered a contraindication for rhinoplasty, as there is no significant increase in the need for revision surgeries in actively smoking patients compared to nonsmokers. While smoking is commonly associated with impaired wound healing and increased risk of infection due to its negative effects on tissue perfusion and immune response, our data demonstrate that these concerns can be managed effectively in the context of rhinoplasty.

Among the 1884 patients included in this study, the revision rates for smokers (3.75%) and nonsmokers (3.29%) were comparable. This finding is particularly notable as it challenges the assumption that smokers are inherently at higher risk for suboptimal surgical outcomes. Despite the known systemic effects of smoking on vascular health and tissue healing, rhinoplasty in smokers appears to result in satisfactory outcomes when proper postoperative care is implemented.

**Figure:**
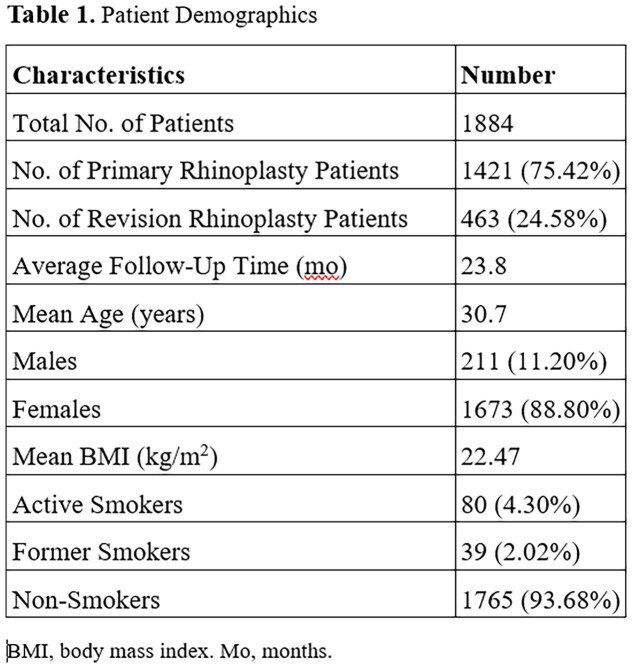

**Figure:**
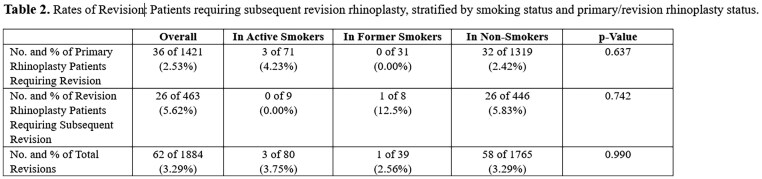

**Figure:**